# Formation of Halogenated Byproducts upon Water Treatment with Peracetic Acid

**DOI:** 10.1021/acs.est.1c06118

**Published:** 2022-03-31

**Authors:** Giulio Farinelli, Marco Coha, Davide Vione, Marco Minella, Alberto Tiraferri

**Affiliations:** † Department of Environment, Land and Infrastructure Engineering (DIATI), Politecnico di Torino, Corso Duca degli Abruzzi 24, 10129 Turin, Italy; ‡ Department of Chemistry, Università di Torino, Via Pietro Giuria 5, 10125 Torino, Italy

**Keywords:** peracetic acid, bromoform, wastewater, halogenated byproducts, oxidation, disinfection, hydrogen peroxide

## Abstract

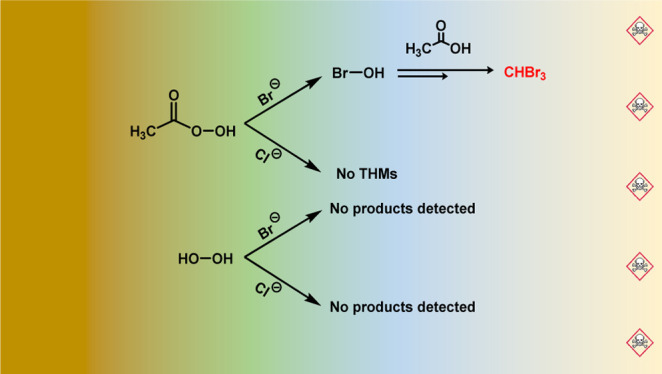

Peracetic acid has quickly gained ground in water treatment over the last decade. Specifically, its disinfection efficacy toward a wide spectrum of microorganisms in wastewater is accompanied by the simplicity of its handling and use. Moreover, peracetic acid represents a promising option to achieve disinfection while reducing the concentration of typical chlorination byproducts in the final effluent. However, its chemical behavior is still amply debated. In this study, the reactivity of peracetic acid in the presence of halides, namely, chloride and bromide, was investigated in both synthetic waters and in a real contaminated water. While previous studies focused on the ability of this disinfectant to form halogenated byproducts in the presence of dissolved organic matter and halides, this work indicates that peracetic acid also contributes itself as a primary source in the formation of these potentially carcinogenic compounds. Specifically, this study suggests that 1.5 mM peracetic acid may form around 1–10 μg/L of bromoform when bromide is present. Bromoform formation reaches a maximum at near neutral pH, which is highly relevant for wastewater management.

## Abstract 2

The peracetic acid reagent contributes itself to hazardous bromoform formation when employed to oxidize/disinfect waters containing bromide: its use requires careful evaluation.

## Introduction

The antimicrobial properties of peracetic acid (PAA) were reported as far back as 1902,^1^ and over the last century PAA has been recognized as an extremely efficient disinfectant toward a wide spectrum of microorganisms.^2^ This feature has promoted the application of PAA in many industrial fields, such as food and beverages, healthcare, textiles, as well as pulp and paper industries.^1,2^ In the early 1980s, PAA also gained a position in the wastewater treatment industry, where it found the most fertile market.^2−5^ This success is mostly due to the increased mandate to reduce chlorine usage, which is associatedwith the formation of carcinogenic chlorinated byproducts.^6−8^ The larger oxidation potential compared to chlorine and chlorine dioxide and the higher antimicrobial efficiency with respect to H_2_O_2_ explain the increasing demand of PAA in the water disinfection field.^2,9^ However, the chemical behavior of PAA in an aqueous medium is complex since this compound is added in the form of a quaternary equilibrium mixture containing acetic acid (AA), H_2_O_2_, and PAA. Indeed, PAA is synthesized according to the reaction between AA and H_2_O_2_, catalyzed by sulfuric acid^10,11^
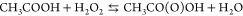
1

Both AA and H_2_O_2_ play major roles in the disinfection process when the PAA mixture (PAAM, i.e., the mixture of AA, H_2_O_2_, and PAA at equilibrium) is employed. AA can potentially allow bacteria to regrow.^12^ Conversely, H_2_O_2_ can potentially compete in the disinfection process acting as a primary disinfectant.^13^ For these reasons, the mode of disinfection of PAA has not been entirely clarified so far,^2^ and the scientific community has encountered difficulties describing the PAAM disinfection byproducts (DBPs) and their mechanism of generation. Initially, no halogenated DBPs, such as trihalomethanes (THMs), were reported in PAA-treated surface water.^14^ Shortly after, researchers corroborated those initial results and reported the formation of aldehydes in the order of μg/L.^15−17^ On the contrary, later studies remarked that in the presence of chloride and phenol, PAAM is able to generate chlorophenols. The process might possibly follow a radical mechanism since it was proven that PAAM could not oxidize chloride directly to hypochlorite.^18^ However, discrimination of the relative behavior of PAA and H_2_O_2_ was not carried out in those studies.

Despite the observation that PAAM can hardly oxidize chloride [the kinetic constant of the reaction PAA + Cl^–^ is very low, *k* = (1.47 ± 0.58) × 10^–5^ M^–1^ s^–1^], this mixture has been shown to oxidize bromide to hypobromous acid and to potentially generate brominated byproducts (the kinetic constant of the reaction PAA + Br^–^ is 0.24 ± 0.02 M^–1^ s^–1^).^2,19,20^ Bromide is present in virtually all water sources at concentrations ranging from ∼10 to 1000 μg/L in fresh waters and of roughly 67 mg/L in seawater.^21^ Therefore, the role of bromide in the PAAM system may be significant. Shah et al. gained insight into the chemical behavior of PAAM in saline waters and discriminated the reactivity of H_2_O_2_ from that of PAA, thus noting the formation of mostly bromoform and haloacetic acids.^19,20^ The authors ascribed the formation of halogenated byproducts to the reaction between hypobromous acid (formed by PAA and bromide) and dissolved organic matter (DOM).^19^ The formation of halogenated byproducts in the absence of DOM and the DBP formation mechanism have not been discussed or explained so far. Indeed, if hypobromous acid can react with DOM, one may be prone to hypothesize that it might also react with AA present in PAAM or formed after the oxidation of bromide by PAA or even with the acetyl group of PAA itself. In other words, the acetyl groups of PAA or AA might be the organic substrate for a bromination reaction.

This study investigates the ability of PAAM and PAA to work as sources of halogenated compounds in the presence of halides, even in the absence of DOM or organic matter other than the AA from PAAM, which is relevant for several applications in the water treatment field. Moreover, specific experiments are discussed that provide a likely interpretation of the reaction mechanismand give insight into the role of pH and reactant concentration. The chemical behavior is studied in both simplified synthetic waters and in real contaminated water currently treated with PAAM in a wastewater treatment plant. Therefore, the main objectives of this work are (i) understanding when and how to safely dose PAAM in an aqueous effluent, which is consequential for practical PAA applications as well as (ii) proposing a mechanistic interpretation of the prevailing reaction. Also, a protocol for the quantitative assessment of the relative amount of PAA and H_2_O_2_ in the PAAM mixture is proposed that combines two established methods for H_2_O_2_ quenching and for the quantification of total oxidants, respectively.

## Materials and Methods

### Chemicals and Real Contaminated Water

The PAA mixture was purchased from Acros Organics (Rodano, MI, Italy). The solution of hypobromous acid was provided by Farm Srl (Guidonia-Montecelio, RM, Italy). All other reagents were purchased from Sigma-Aldrich (Milan, Italy) and used as received without any further purification steps. Groundwater receiving leachate from a phosphogypsum landfill was directly obtained from the pumping wells in a contaminated site in the south of Italy and used as is. The main characteristics of this real contaminated water are summarized in Table S1 of the Supporting Information (hereinafter SI). A significant concentration of microalgae [(1.76 ± 0.6) × 10^6^ cells/mL] as well as DOM (TOC = 58 ± 12 mg^C^/L) was present in this water (pH 2.8), which did not contain halogenated organic compounds at detectable concentrations (see Table S2 of the Supporting Information). This water is currently treated in a wastewater treatment plant before discharge into the environment, including addition of PAA mixture within the treatment train. Unless otherwise stated and except for the experiments performed with the real contaminated water, all experiments were performed in type I water obtained from a Millipore-Merck system (TOC ≤ 2 ppb).

### Reaction Procedures

All reactions in this study were conducted in 10 mL solution in hermetically closed vials protected from the action of light with an aluminum foil. Except for experiments conducted in the real contaminated groundwater, the pH was equal to 5.2 due to the presence of AA, and it was measured both at the beginning of the reaction and at the end of the process: no pH variation was detected following the reaction. The final samples for the analysis of CHBr_3_ were taken at different times for each experiment (typically, after 30 to 60 min) and, specifically, when the curve C/C_0_ of the oxidant (which includes the oxidant content of PAAM and possible oxidizing species formed in solution) versus time reached a plateau (see Figure S1 in the Supporting Information).

### Analytical Methods

**Figure 1:**
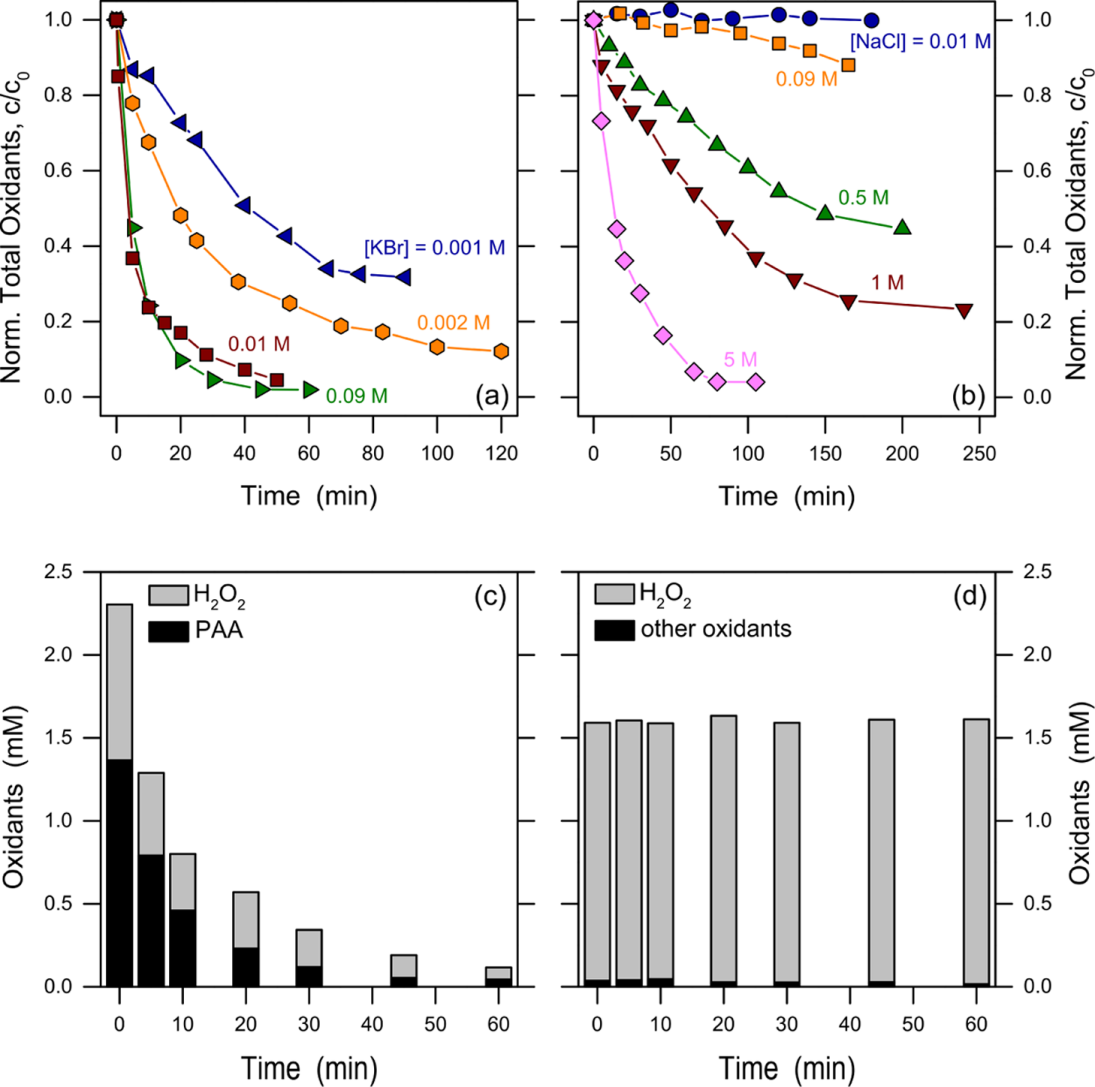
Consumption of total oxidants in the presence of halides. (a) Consumption in a solution of PAAM in which the initial PAA is 1.5 mM with KBr at different concentrations. (b) Consumption in a PAAM solution in which the initial PAA is 1.5 mM with NaCl at different concentrations. Lines connecting the data points are only intended as guides for the eye. (c) Consumption of H_2_O_2_ and PAA in a solution of PAAM in which the initial PAA is 1.5 mM and KBr is 10 mM. (d) Consumption in a solution of H_2_O_2_ (1.5 mM) and KBr (10 mM). In (c,d), a solution of catalase (6.7 mg/L) added to the samples withdrawn for the analysis from the reaction batch for each time step allowed for discrimination between PAA and H_2_O_2_.

Halomethane formation in synthetic water samples was determined with a GC–MS analyzer (HP 6890 Series GC system equipped with a HP 5973 mass selective detector). A sample aliquot (1 mL) was taken when the total oxidant *C*/*C*_0_ ratio was near 0 or otherwise consistently low (total oxidant is intended as the sum of all possible oxidant species in the target sample, including PAA and H_2_O_2_); see Figure 1. The sample was diluted 1:50 in a 50 mL volumetric flask containing an aqueous solution and 0.5 g of NaOH. Halomethanes were analyzed through a purge and trap system (P&T Tekmar LSC 2000 coupled to an Entech 7000 focuser) in a 3 mL volume aliquot. The purge time was 11 min, followed by 4 min of purge drying. Then, the volatile compounds were cryofocused at −200 °C. The focusing program lasted 3.5 min, after which the sample was injected into the GC system. An Agilent CP-SIL 5 CB column (length 60 m, internal diameter 0.32 mm, film width 1 μm) was used for the chromatographic separation. The carrier gas was 6.0-grade helium (Sapio, Italy). The injector temperature was 280 °C, and the oven temperature program of the chromatographic system was 35 °C from 0 to 5 min, 5 °C/min ramp up to 140 °C, 15 °C/min ramp up to 240 °C, and 15 min at 240 °C (total run time 47.7 min). The MS detector was operated in the scan mode. The monitored halomethanes were bromomethane (*t*_r_ = 5.16 min, peaks at 15 and 94 *m*/*z*), dibromomethane (*t*_r_ = 13.60 min, peaks at 93 and 174 *m*/*z*), tribromomethane (*t*_r_ = 21.54 min, peaks at 91 and 173 *m*/*z*), chloromethane (*t*_r_ = 4.44 min, peaks at 15 and 50 *m*/*z*), dichloromethane (*t*_r_ = 7.25 min, peaks at 49 and 84 *m*/*z*), trichloromethane (*t*_r_ = 10.27 min, peaks at 47 and 83 *m*/*z*), and dibromochloromethane (*t*_r_ = 17.76 min, peaks at 48 and 129 *m*/*z*). The limit of detection (LOD) of this method is approximately 500 ng/L. The halomethanes generated in the real contaminated groundwater upon PAAM addition were analyzed at a private external laboratory [Chelab srl—Merieux Nutrisciences, Volpiano (TO), Italy] using the method EPA 5030 C 2003 + EPA 8260 D 2018, which allows determination of the byproducts listed in Tables S2 and S3 of the Supporting Information. Note that this method does not include haloacetonitriles. All analyses for the determination of the oxidant concentration (i.e., H_2_O_2_ or the sum of PAA and H_2_O_2_) were spectrophotometrically performed at an analytical wavelength of 350 nm (vide infra).

### PAA Solution Characterization

The PAAM mixture consists of the following nominal partial concentrations, as indicated by the manufacturer: PAA 34–39%; AA 46–55%; H_2_O_2_ 11–15% (i.e., PAA 5.05–5.79 M; AA 8.66–10.35 M; H_2_O_2_ 3.65–4.98 M). An acid-base titration with standardized NaOH of the PAAM solution with phenolphthalein as the indicator allows verification of the concentration of AA. The titration was performed after 50× dilution, and the result was a concentration of AA equal to 9.81 M, which is consistent with the data provided by the supplier. Please note that PAA does not compete with such titration because its titration conditions are not reached (the pKa of PAA is 8.2, while the pKa of AA is 4.75).^22^ The triiodide method was used to quantify the concentration of total oxidants in the PAA mixture, which mostly include PAA and H_2_O_2_. The method was adapted from previous studies, and an explanation follows.^19^ Three mL of sample (2.9 mL of MilliQ water and 0.1 mL of PAAM, pH 5.2), 1 mL of solution A (66 g/L KI; 2 g/L NaOH; 0.2 g/L ammonium heptamolybdate), and 1 mL of solution B (40 g/L potassium hydrogen phtalate) were added in a 5 mL volumetric flask. Then, the absorbance of this solution was measured at the wavelength of 350 nm. The total concentration of oxidants ([PAA] + [H_2_O_2_]) in the PAAM was estimated as 9.3 M in accordance with the data provided by the supplier (vide supra).

In the experiments performed to understand the stability of PAAM in the presence of halides, the individual concentrations of PAA and H_2_O_2_ were measured by adding the enzyme catalase from bovine liver to the samples withdrawn for the analysis at different time steps from the reaction batch. Catalase was used to decompose H_2_O_2_, and the triiodide method was used to quantify the residual oxidant, namely, PAA. The difference between the total concentration of oxidants and the PAA concentration allows for finally estimating the H_2_O_2_ concentration. This approach is enabled by the fact that the equilibrium reaction has very slow interconversion kinetics, and thus PAA takes time to regenerate H_2_O_2_ degraded by catalase.^10,11^ Catalase only reacts with H_2_O_2_, even if other peroxides are present.^23,24^ To quantify the correct amount of catalase (catalase/H_2_O_2_ 4:1 wt.) and the time (5 min) needed to decompose H_2_O_2_, preliminary experiments were performed, and the results are shown in the Supporting Information (Figure S2). Specifically, Figure S2a shows the volume of a solution of catalase (6.7 mg/L) needed to completely decompose H_2_O_2_ (1.5 mM). Figure S2b shows the time needed to completely decompose H_2_O_2_, namely, 5 min. Figure S2c shows the effect of catalase on H_2_O_2_ in a solution where PAAM was dosed to obtain PAA 1.5 mM in solution. These results suggest that [H_2_O_2_] = 0.66 × [PAA] in PAAM. The results additionally imply that a slightly acidic pH does not affect the function of catalase; these latter experiments were performed at pH 5.2, that is, the natural pH of the PAAM solution after dilution, which was adopted in all experiments of this study, unless otherwise stated.

## Results and Discussion

### Stability of PAAM in the Presence of Halides

Figure 1 shows the consumption of total oxidants present in PAAM (dosed to achieve 1.5 mM PAA in solution) at varying concentrations of KBr or NaCl. When bromide was present, the total oxidants were consumed rapidly even at 1 mM KBr (Figure 1a). This process reached an asymptote (vs [KBr]) at 10 mM KBr since only a marginal increase in the degradation kinetics of total oxidants was observed when the concentration of KBr was further increased up to 90 mM. This observation is consistent with previous studies, which proved the ability of PAA to oxidize bromide to hypobromous acid in water.^19,20^ On the contrary, the results reported in Figure 1b indicate that 10 mM NaCl did not have significant effects on the stability of PAAM. The consumption of total oxidants became visible with 90 mM NaCl, then proceeding more and more rapidly at higher concentrations.

The need for a high concentration of chloride, namely, 5 M, to produce the same consumption effects observed in the presence of 10 mM KBr suggests that the reactivity of chloride toward oxidants contained in PAAM is low from a kinetic standpoint and/or that small and hardly detectable bromide impurities contained in NaCl might be responsible for the degradation. PAA should be able to oxidize both bromide and chloride from a thermodynamic standpoint (*E*_0_ PAA/AA = 1.81 V vs NHE at pH 7;^25^*E*_0_ Br^–^/Br_2_ = 1 V vs NHE; *E*_0_ Cl^–^/Cl_2_ = 1.36 V vs NHE),^9^ but our data suggest that the process involving chloride takes place with perceptible kinetics in the absence of other forms of dissolved organics and/or catalysts, only at high chloride concentrations (Cl^–^ ≥ 0.5 M).^26^

Based on the kinetic data reported by Shah et al.,^19^ we compared the expected rate constants for the reaction of PAA with bromide and chloride at different concentration values of the two anions (see Text S1 in the Supporting Information). The kinetic data suggest that PAA disappearance in the presence of chloride might be due to small bromide impurities occurring in NaCl, rather than to chloride itself. Comparison of the experimental kinetics (Figure 1a,b) suggests that these impurities might amount to 0.2%, which is compatible with the purity degree of our reagent. Interestingly, a study by Crathorne et al. supports our results about poor reactivity of chloride by discussing the absence of chlorinated byproducts when PAA was added to a solution of humic acid enriched with chloride.^27^ Therefore, the process of PAA/PAAM consumption would involve bromide rather than chloride in practically all conditions, ranging from high-salinity streams to typical wastewaters and to all bromide-rich aqueous streams.^28^

As far as the oxidant consumption pathway in the presence of Br^–^ is concerned, our data suggest that H_2_O_2_ does not react with bromide to generate hypobromous acid; see Figure 1d. This result is consistent with previous literature, which indicated that the oxybromination reaction (HBr + H_2_O_2_ → HOBr + H_2_O) needs a catalyst to occur (the process can also be triggered in acidic solution, where the catalyst would be H^+^).^29,30^ The consumption of H_2_O_2_ observed in Figure 1c can be justified by virtue of the reaction reported in the literature, according to which the conjugate base of H_2_O_2_ (HO_2_^–^) reacts with hypobromous acid to produce bromide.^19^ In turn, the occurrence of HOBr in the system would be accounted for by the well-known oxidation process of Br^–^ by PAA^19^
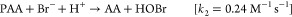
2
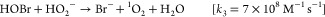
3

Considering reactions 2, 3 with p*K*_a_ = 11.6 for H_2_O_2_^19^ and pH 5.2 as per the conditions of our experiments, one has that the rate constant of reaction 3 in our experimental conditions would be ∼10^3^ times higher than the rate constant of reaction 2. Therefore, reaction 2 can be considered as the rate-determining step of the process. From the values of *k*_2_ and [Br^–^], one gets a pseudo-first-order lifetime of PAA in reaction 2 in the order of ∼5 min that should be the same as the lifetime of H_2_O_2_. These predictions are in good agreement with the time trends of PAA and H_2_O_2_ reported in Figure 1c (see also Figure S4 in the Supporting Information). Moreover, according to reactions 2, 3, Br^–^ would have a catalytic role in inducing PAAM consumption. In contrast, PAA was stable over a time scale of a few hours in the absence of bromide.

### Formation of THMs

**Figure 2:**
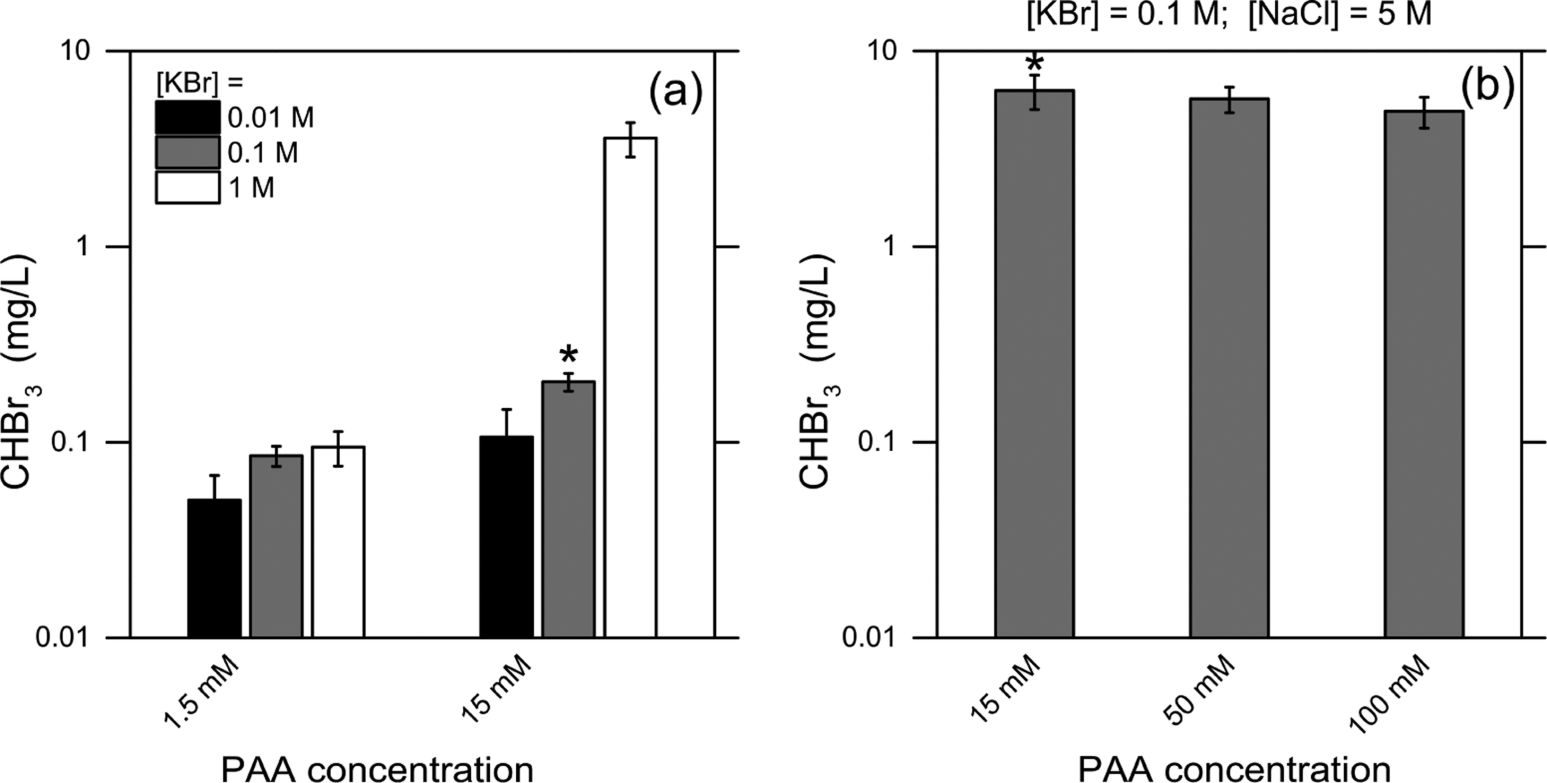
Formation of CHBr_3_ in the presence of PAA and halides. (a) CHBr_3_ measured in solution as a function of KBr (0.01; 0.1; 1 M) and PAA (1.5; 15 mM). (b) CHBr_3_ measured in solution in the presence of PAA, at different initial concentrations of PAA (15; 50; 100 mM) in a solution containing KBr (0.1 M) and NaCl (5 M). The star symbol (*) over the bars indicates different experiments but performed at equivalent concentrations of PAA + KBr (a) without and (b) with NaCl. The samples for analysis were taken at the time when the corresponding depletion profile of the total oxidant concentration reached a plateau (typically, 30 to 60 min depending on the experiment; see Figure S1 in the Supporting Information). All tests were run in duplicate, with results always within 20% for the same conditions.

Bromoform (CHBr_3_) was the only halomethane detected in the presence of PAA + NaCl + KBr, regardless of the initial concentration of the reactants (see Figure 2). No other halomethanes were detected upon treatment of the synthetic waters with PAAM. Experiments were then performed at different PAAM doses and by varying the PAA/Br^–^ ratio. Figure 2a presents the concentration of CHBr_3_, formed upon PAAM addition to obtain a PAA concentration of 1.5 or 15 mM, at varying levels of KBr (0.01–1 M).

The observed increase of bromoform concentration following the increase of PAA and/or bromide concentration corroborates the results discussed above and suggests that PAA (or its derivatives including AA) and bromide (or its derivatives) are the species responsible for CHBr_3_ generation (with yields in the range of 0.03–0.08% of the initial PAA). Note that an increase of KBr from 0.01 to 1 M entails an increase of 2 orders of magnitude in the ionic strength, which might potentially have an impact on the reaction. However, very little increase in bromoform formation was observed when passing from 0.01 to 1 M KBr in the presence of 1.5 mM PAA (Figure 2a), thereby suggesting that ionic strength has a limited role in the process.

While previous studies have discussed the formation of brominated byproducts upon oxidation with PAA in the presence of other organic components acting as substrates for the bromination reaction,^2,7,18−20,27,31,32^ this work provides evidence for the role of PAA itself (or its derivatives) as a substrate for CHBr_3_ generation. The data of Figure 2b further corroborate the low activity of chloride toward PAA consumption and halomethane generation. By increasing the concentration of PAA ranging from 15 to 100 mM in a solution with KBr 0.1 M and NaCl 5 M, bromoform remained the only detected halogenated organic byproduct. However, when comparing the results of Figure 2a,b, a significantly higher concentration of bromoform was formed in the solution prepared by dissolving both KBr and NaCl (Figure 2b) with respect to that containing only KBr (Figure 2a) at the same concentration values of PAA and KBr. It is important to mention that no bromoform or chloroform was detected when PAA at 15, 50, or 100 mM was present in a solution of NaCl 5 M. The higher formation of bromoform in Figure 2b might suggest cooperative phenomena among bromide and chloride when simultaneously present in solution (note that possible bromide impurities in 5 M NaCl could not produce 0.1 M bromide in solution). HOBr undergoes equilibrium reactions and may react with chloride to form a host of different chlorinating and brominating agents (e.g., BrCl, Br_2_, BrOCl, and Br_2_O), as noted in previous studies.^33−35^ Many of these species may be more reactive than HOBr and HOCl in undergoing electrophilic substitution reactions. Overall, the evidence discussed so far frames one component of PAAM, namely, PAA or AA, as the organic substrate consumed to form CHBr_3_.

### Reaction Mechanism and Prevailing Species

To gain insight into the previously described phenomenon, it is crucial to further define the prevailing species involved in the process. Previous studies have widely proven the ability of PAA to oxidize bromide, generating hypobromite and AA.
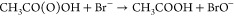
4

**Figure 3:**
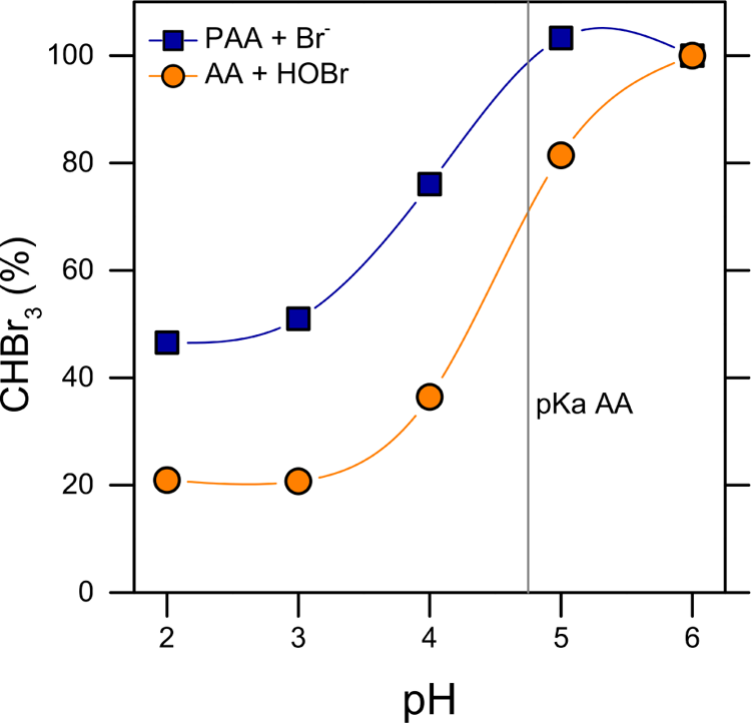
CHBr_3_ produced in solutions of varying pH, starting with (blue) a solution containing PAA (1.5 mM) and KBr (10 mM) and (orange) a solution containing AA (2.8 mM) and HOBr (2 mM). The data for the two systems are normalized by the CHBr_3_ concentration detected at pH 6. The vertical line indicates the p*K*_a_ value for AA. The samples for analysis were taken at the time when the corresponding depletion profile of the total oxidant concentration reached a plateau. Lines connecting the data points are only intended as guides for the eye.

The results reported in Figure 3 suggest the participation of AA in the process. The formation of bromoform was monitored as a function of pH (ranging from 2 to 6) in two different systems. Note that CHBr_3_ was the only byproduct detected in these experiments as well. In the first system, PAA and bromide reacted at the optimized PAA/Br^–^ ratio for the consumption of PAA, namely, [PAA] = 1.5 mM and [KBr] = 10 mM; see Figure 1a. The second system was instead obtained by directly dissolving the products of reaction 4, namely, AA and hypobromous acid. AA was dosed according to its percentage in PAAM when PAA is 1.5 mM, that is, [AA] = 2.8 mM. [HOBr] = 2 mM was chosen to be a bit higher than the amount of HOBr that can be formed in the presence of 1.5 mM PAA. The limiting reagent in the reaction scheme is AA, and addition of excess HOBr serves the purpose of verifying the ability of AA to participate in the reaction to give rise to CHBr_3_.

The system AA-HOBr generated bromoform with analogous pH trend as the PAA–Br^–^ system, which suggests that the same reaction mechanism likely occurred in the two cases. Moreover, the flex point of the curves lied around the pKa value of AA (4.75). These results suggest that AA is the organic substrate responsible for the formation of bromoform when PAA reacts with bromide and that bromoform formation is higher when AA is deprotonated. According to the results summarized in Table 1, it is reasonable to conclude that HOBr is directly involved in the process. In a system containing AA, H_2_O_2_, and KBr, no bromoform was detected as oxybromination needs a catalyst.^29^ On the contrary, AA and HOBr together promoted the formation of bromoform, the concentration of which increased with increasing HOBr concentration (see Table 1).

**Table 1:** Formation of CHBr_3_ in Solutions Consisting of AA (2.8 mM), H_2_O_2_ (1.5 mM), and KBr (10 mM); AA (2.8 mM) and HOBr at Different Concentrations (2; 10 mM)^a^

AA (mM)	HOBr (mM)	H_2_O_2_ (mM)	KBr (mM)	CHBr_3_ (mg/L)
2.8		1.5	10	<LOD
2.8	2			1.01 ± 0.05
2.8	10			3.98 ± 1.54

*AA (^13^C-1) (mM)	KBr (mM)	PAA (mM)	*CHBr_3_/CHBr_3_	total CHBr_3_ (mg/L)
10	10	1.5	0.05	4 ± 0.6

a Additionally, relative formation of the labeled bromoform (*CHBr_3_) in a solution consisting of: 10 mM labeled AA (*AA, ^13^C-1), KBr (10 mM), and PAA (1.5 mM). LOD ∼ 500 ng/L.

In conclusion, we surmise that AA and HOBr (or other brominating electrophile agents) are the most probable species that are responsible for the generation of bromoform in the system PAAM–Br^–^. Tests were also performed in the presence of labeled acetic acid (^13^C-1 AA): here, an enriched fraction of labeled bromoform was detected at the end of the reaction (see Table 1), further corroborating that AA is directly involved in the process. A possible explanation for the relatively low percentage (<5%) of labeled bromoform observed as a reaction product is as follows: the initial reaction 2 between PAA and bromide would yield nonlabeled AA and HOBr, and the two species after formation would be initially surrounded by a cage of water molecules. Therefore, the initial reaction between AA and HOBr (the first step of bromoform formation, vide infra) would be favored over diffusion of HOBr out of the solvent cage, where it could react with labeled *AA occurring in the solution bulk.

**Scheme 1:**
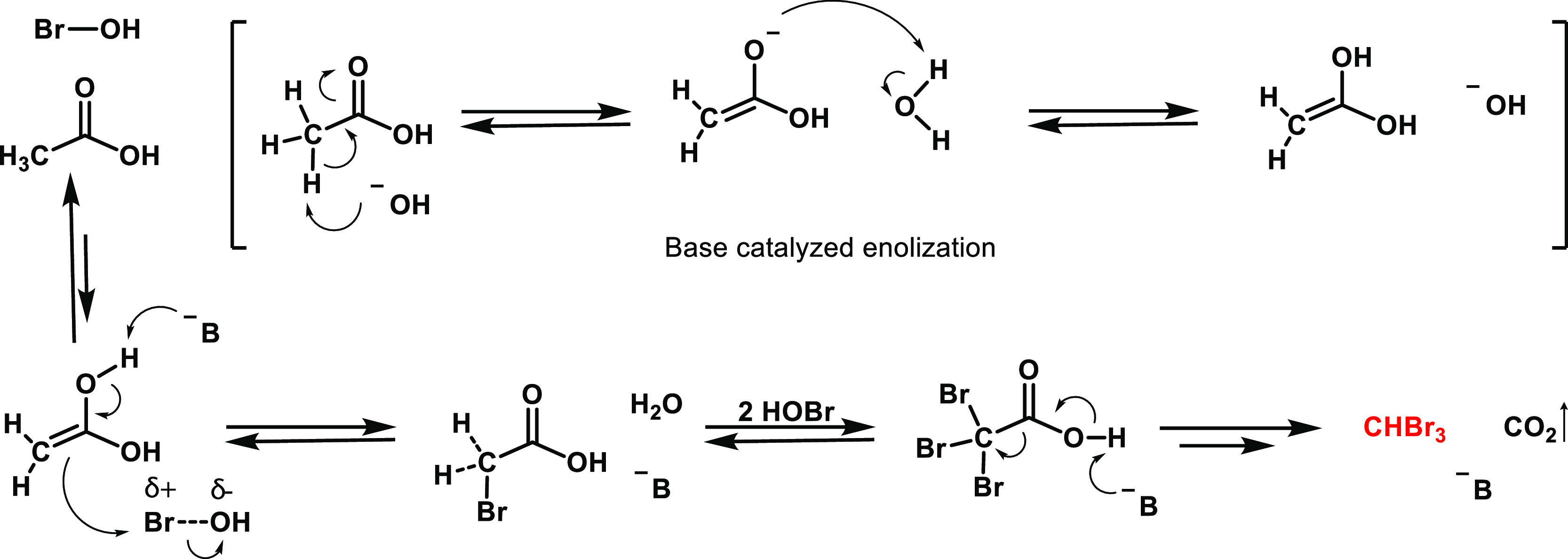
Rationalization of the Possible Reaction Mechanism Causing the Formation of CHBr_3_ in the Presence of PAAM and Br^–^ “B^–^” represents a generic base in solution (such as OH^–^ or acetate). The arrow sign toward the final step of bromoform formation signifies that there could be other intermediate reaction steps, which are not included in the scheme.

In this framework, we surmise that the α-bromination of carboxylic acids (known as Hell-Volhard-Zelinsky reaction) is the likely mechanism of bromoform formation from AA. Scheme 1 shows the mechanism of the reaction reported for the first time between 1880 and 1887.^36,37^ This reaction occurs in aqueous mediums owing to keto-enolic tautomerization, which can also take place with carboxylic acids, as well as esters and amides, and not solely with ketones.^38^ The enolate would represent the nucleophile that is able to react with the electrophile (e.g., HOBr). Following the first addition of bromine, the monobrominated methyl group becomes more electronegative, and hence it is more prone to accept other two equivalents of bromine to produce CBr_3_^–^, which is a stable exit group. A basic environment can help both the tautomerization process and the electrophilic addition.^34,35^ This rationalization would explain why pH played a role and why higher CHBr_3_ formation occurred in our study when AA was deprotonated. When CBr_3_^–^ exits, it regenerates the basic environment by forming CHBr_3_ + OH^–^. The formation of stable CHBr_3_ breaks the reversible condition of the process and pushes the reaction toward further bromoform formation. Indeed, CHBr_3_ is a typical byproduct of the α-bromination of carboxylic acids.^37^

The results summarized in Table 2 corroborate the hypothesis related to the reaction pathway. According to the Hell-Volhard-Zelinsky mechanism, the formation of CHBr_3_ would be contemplated only when AA is the carboxylic organic substrate. Propionic and butyric acids were thus used as mechanistic probes in order to evaluate the proposed hypothesis. In the presence of propionic or butyric acid, the possible exit groups would be 1,1-dibromoethane and 1,1-dibromopropane, respectively. However, no evidence of CHBr_3_ formation was found, and no traces of 1,1-dibromoethane or 1,1-dibromopropane were detected during tests conducted with the use of propionic or butyric acid instead of AA. Suitable exit groups, namely, CBr_3_^–^ in the case of AA, C_2_H_3_Br_2_^–^ in the case of propionic acid, and C_3_H_5_Br_2_^–^ in the case of butyric acid, should be soluble and stabilized in some way for the reaction to occur. The two latter exit groups, reasonably, would have lower ability to stabilize their negative charge through interaction with the solvent and hence would be less soluble in the aqueous medium and unlikely to occur.

**Table 2:** Formation of CHBr_3_ in Solutions Containing HOBr 2 mM and Different Organic Substrates (AA; Propionic Acid; Butyric Acid) at 2.8 mM Initial Concentration^a^

substrate (2.8 mM)	HOBr (mM)	CHBr_3_ (mg/L)
AA	2	1.01 ± 0.05
propionic acid	2	<LOD
butyric acid	2	<LOD

a LOD ∼ 500 ng/L.

**Scheme 2:**
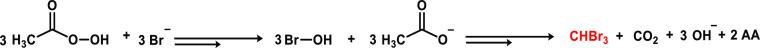
Rationalization of the Steps Occurring to Produce CHBr_3_ in a Solution Containing PAA and Bromide, which Includes the Participation of a Br-Containing Electrophile Species (Exemplified as HOBr) and Acetate/AA Species The arrow signs between steps signify that there could be other intermediate reaction steps, which are not included in the scheme.

Further support for the mechanism discussed here comes from the study by Shah et al., who observed the sole formation of bromoform and brominated acetic acids in an aqueous medium rich in bromide and treated with PAAM, although in the presence of DOM.^20^ The fact that only a slight increase of labeled bromoform was observed in the experiments performed using ^13^C-1 AA (Table 1) implies that PAA is the species that at least initially triggers the formation of bromoform, although the Hell-Volhard-Zelinsky mechanism then involves AA that is formed as a reaction intermediate. This explanation is consistent with the hypothesis of a concerted process, in which all reaction steps occur inside the solvent cage. According to the results of this work, Scheme 2 is proposed to summarize the overall process involved in the formation of bromoform when PAAM and bromide are simultaneously present in solution.

### Implications for PAAM Use in Real Waters

**Figure 4:**
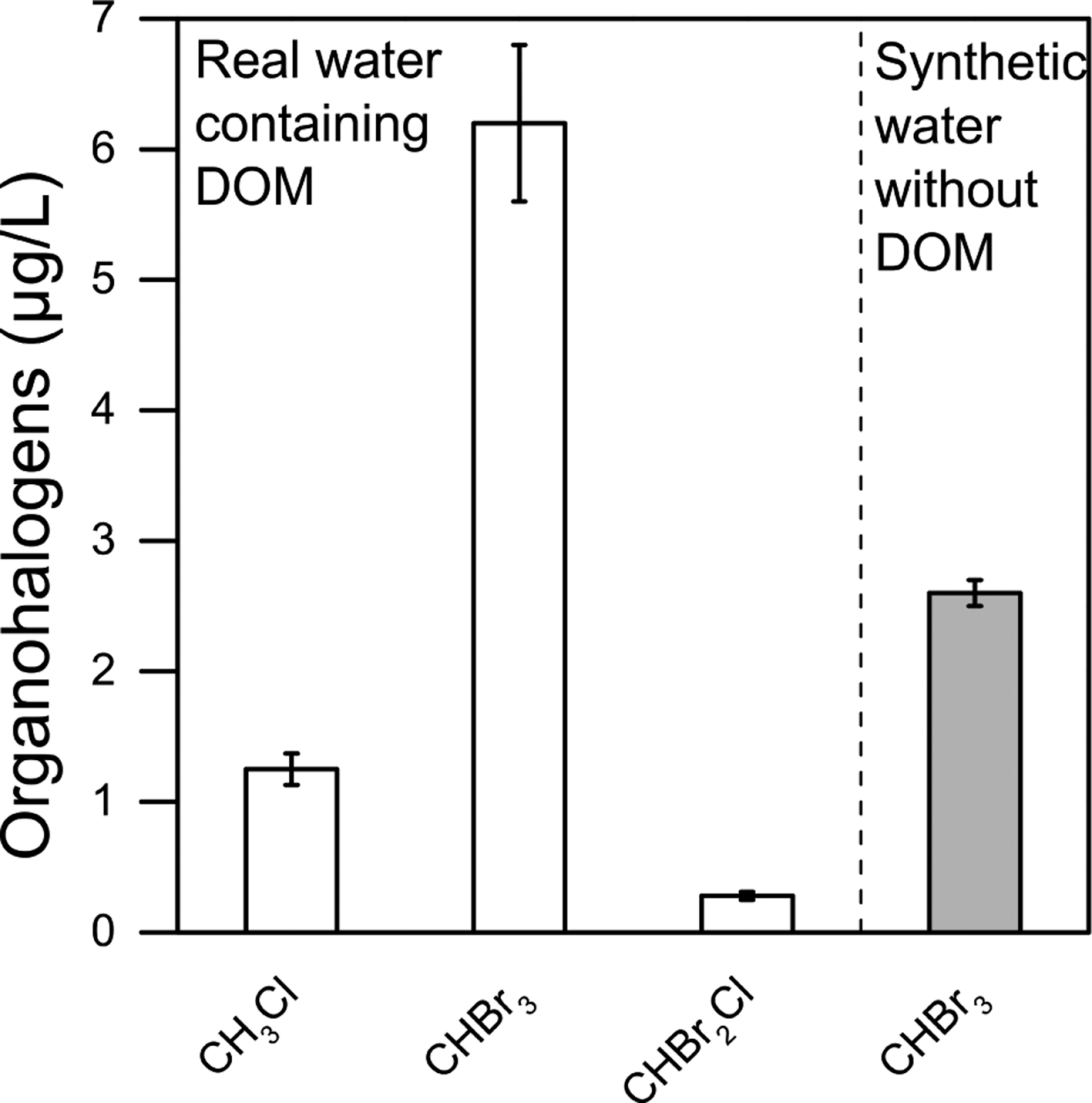
Organohalogens detected upon addition of PAAM in (white bars) real contaminated groundwater (Tables S1–S3) and in (gray bar) synthetic water consisting of the same ionic composition and pH of the real groundwater but in the absence of DOM and other biological materials. The error bars represent standard deviations obtained from three replicates for each test.

In the last decades, the interest in using PAAM in water treatment industry has substantially grown.^1,2,7,15,17^ However, this study suggests that the use of PAAM should be considered carefully in the presence of bromide, even in the absence of natural DOM or other organic compounds in the effluent. Aiming to verify the output of PAAM addition in a real and complex environment, we tested real contaminated groundwater (see Table S1 in the Supporting Information for further details on the matrix composition). The selected matrix contained 6.4 mg/L of bromide (∼80 μM), around 3 g/L of chloride (0.09 M), and ∼58 mg/L of TOC, and the PAA concentration was 1.5 mM upon addition of the oxidant mixture. Figure 4 shows that also in a highly complex solution with multicontamination parameters and in the presence of a high concentration of TOC, bromoform is the main byproduct of the PAAM treatment. As far as the chlorine-containing byproducts are concerned, the kinetic data available in the literature^19^ suggest that in our system, HOCl would be formed from PAA + Cl^–^ at a rate that is ∼15 times lower compared to HOBr formed from PAA + Br^–^. Considering that HOCl reactivity in the haloform reaction is also much lower compared to the reactivity of HOBr, such a process does not appear as a reasonable source of chlorinated compounds. Therefore, other substrates might be involved in the process. For instance, chloromethane is known to be produced through the substitution of chloride on methanol in the presence of metals as catalysts (and similar processes involving other organic compounds might occur in a complex multicontaminated matrix),^39^ while the presence of chloro-dibromomethane may be attributed to the substitution of chloride on bromoform. Note that the results presented in Figure 4 are consistent with what was observed in the wastewater treatment plant, where analyses performed at different times showed the presence of total amount of halogenated compounds between 2.5 and 10.9 μg/L following addition of PAAM (equivalent to a concentration of PAA equal to 1.5 mM), with the major fractions represented by CHBr_3_ (45–60%) and CH_3_Cl (20–45%).

The results summarized in Figure 4 also show that when the same amount of PAAM dosed in the real groundwater (1.5 mM) was spiked to synthetic groundwater with the same ionic composition (including 80 μM Br^–^ and 0.09 M Cl^–^) and same pH (2.8; achieved by addition of HCl), but in the absence of DOM or other biological materials, a significant albeit lower concentration of bromoform was detected in solution (2.6 μg/L). The value of bromoform detected in the synthetic groundwater amounted to a relevant percentage of the value detected in the real groundwater. Therefore, in the specific real effluent investigated in this study, PAA may have had a non-negligible role as a source of bromoform upon addition of PAAM. While DOMs with different chemical compositions would likely be characterized by a range of activity toward bromoform formation, the results presented here suggest that even in a complex matrix containing a substantial concentration of DOM, PAA may play a non-negligible role in the production of brominated byproducts. While the relative contribution of different carbon sources may vary, an important implication of this study is that the formation of bromoform in PAA-based disinfection is independent of the presence of other kinds of organic matter in the water effluent. Therefore, a certain amount of bromoform would be produced even in a virtually organic-free effluent, which is corroborated by the results presented in Table 1 where *AA was a source of bromoform coherently with Scheme 2 proposed in this study. The results obtained with synthetic waters and presented in Figure 4 would imply that bromoform generation related to PAA should be more significant at near-neutral pH, which is even more relevant for the majority of other water and wastewater effluents.

Finally, this study confirms the slow oxidative activity of H_2_O_2_ toward halides to generate more reactive species, such as HOBr or HOCl, and hence the low or nil activity of the latter species toward DOM or other carbon sources when H_2_O_2_ is the disinfectant.^29^ This phenomenon would thwart the generation of THMs in the presence of DOM during a disinfection process mediated by hydrogen peroxide. Indeed, in a previous study, H_2_O_2_ was tested as a potential disinfectant in the same real groundwater tested in this work, showing no traces of THMs upon treatment,^40^ different from PAA (see Figure 4).

## Supporting Information Available

Depletion profile of total oxidants when PAAM was dosed in solutions containing different concentrations of KBr and/or NaCl; relative H_2_O_2_ concentration in the solution following addition of catalase; comparison of the reaction kinetics of PAA with chloride and bromide; fit of the experimental data shown in Figure 1c; indication of unlikelihood of the exit of the halogenated group in the case of long-chain carboxylic acids, according to a Hell-Volhard-Zelinsky mechanism; characteristics of the real contaminated groundwater; and list and quantification of halomethanes in the real contaminated groundwater before treatment and following treatment with PAA 1.5 mM (PDF)
