# Supplementary material for: Formation of Halogenated Byproducts upon Water Treatment with Peracetic Acid
Source: Environ Sci Technol. 2022 Mar 31;56(8):5123–31. doi: 10.1021/acs.est.1c06118 (PMC9022431; doi:10.1021/acs.est.1c06118)
Supplement: Supplementary file 1 — es1c06118_si_001.pdf [file es1c06118_si_001.pdf]

Supporting Information for

Formation of Halogenated By-products upon Water Treatment with Peracetic Acid

Giulio Farinelli,*[,†,1] Marco Coha,[†,1] Davide Vione,[§] Marco Minella,[§] Alberto Tiraferri*[,†]

[†]Department of Environment, Land and Infrastructure Engineering (DIATI), Politecnico di Torino, Corso Duca degli Abruzzi 24, 10129, Turin, Italy

[§] Department of Chemistry, Università di Torino, Via Pietro Giuria 5 - 10125 Torino (Italy)

* Corresponding Authors:

Giulio Farinelli. Email: giulio.farinelli@polito.it

Alberto Tiraferri. Email: alberto.tiraferri@polito.it

[1] G.F. and M.C. contributed equally to this paper.

This document contains 12 pages, which include four figures (pages S2, S3, S4, S6), one reaction scheme (Scheme S1 on page S7), three tables (pages S8-12), and one text (pages S4-5) discussing the comparison of the reaction kinetics of PAA with chloride and bromide.

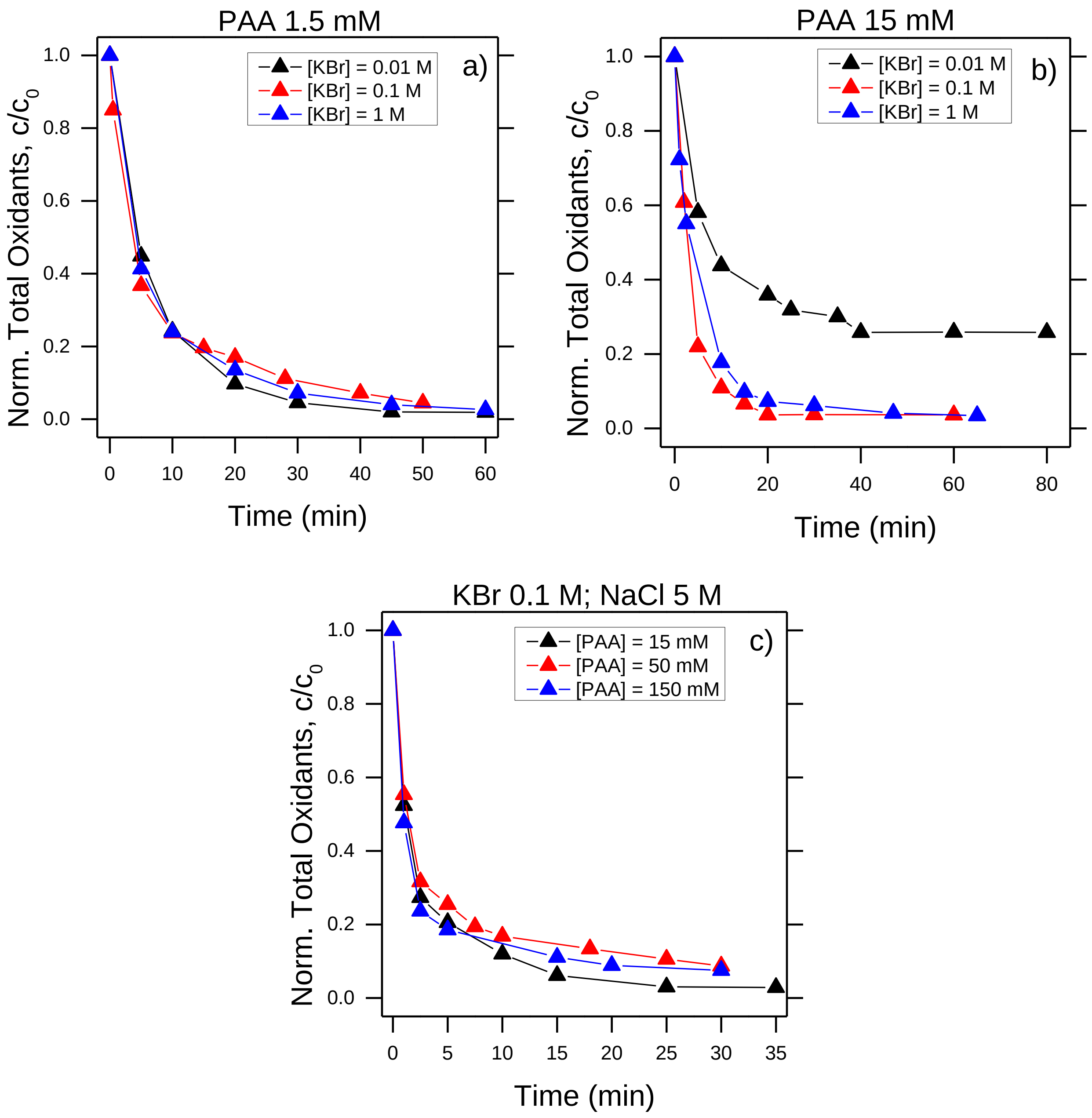

Figure S1. (a) Depletion profile of total oxidants when PAAM was dosed in order to reach the concentration of PAA 1.5 mM in solution at different concentrations of KBr. (b) Depletion profile of total oxidants when PAAM was dosed in order to reach the concentration of PAA 15 mM in solution at different concentrations of KBr. (c) Depletion profile of total oxidants when PAAM was dosed in a solution of KBr 0.1 M and NaCl 5 M, in order to reach the concentrations of PAA equal to 15, 50, and 150 mM.

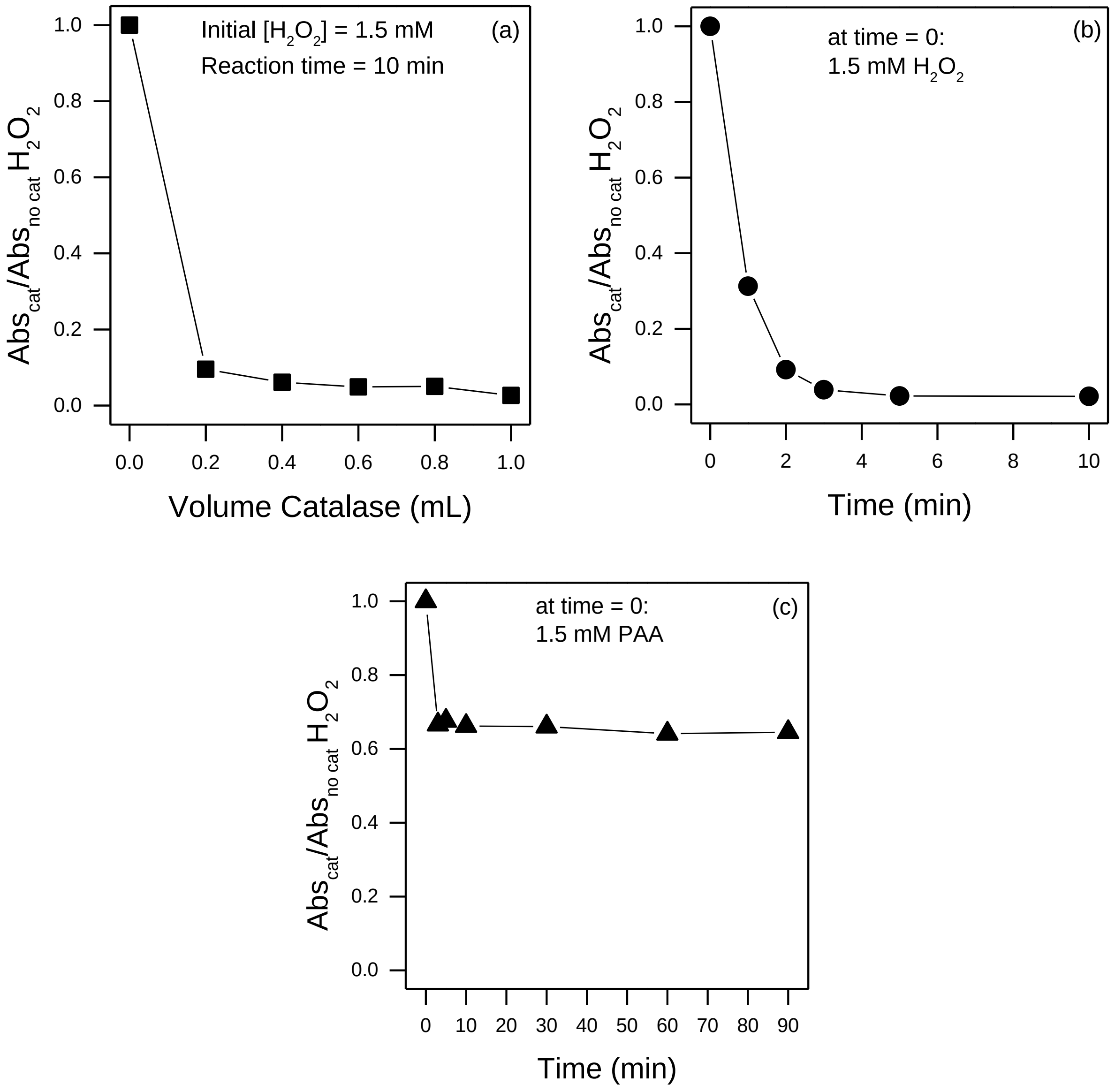

Figure S2. Relative H_2O_2 concentration following addition of catalase, expressed as optical absorbance upon addition of catalase with respect to that in the absence of catalase. (a) Effect of concentration of catalase on relative H_2O_2 concentration. The analyses were carried out with UV-Vis spectrophotometer after 10 min. (b) Relative H_2O_2 concentration as a function of time upon addition of catalase at catalase:H_2O_2 ratio of 4:1 (wt/wt) in 1.5 mM H_2O_2 solution. (c) H_2O_2 concentration as a function of time upon addition of catalase in 1.5 mM PAA solution. Here, catalase was dosed at catalase:H_2O_2 4:1 ratio (wt/wt). The pH of the solutions was 5.2. The graphs imply that a slightly acidic pH does not affect the activity of catalase. Abs_{cat} and $Abs_{no\ cat}$ refers to the absorbance of the solution with catalase and the solution without catalase, respectively.

Text S1. Comparison of the reaction kinetics of PAA with chloride and bromide

According to literature data, the following reactions are involved in the degradation of peracetic acid (PAA) in the presence of chloride and bromide, respectively:

$$PAA + Cl^- + H^+ \rightarrow AA + HOCl \quad [k_1 = 1.5\times10^{-5}\ M^{-1}\ s^{-1}] \quad (S1)$$

$$PAA + Br^- + H^+ \rightarrow AA + HOBr \quad [k_2 = 0.24\ M^{-1}\ s^{-1}] \quad (S2)$$

Then, HOCl and HOBr produced in reactions (S1, S2 respectively) take part to the degradation of H_2O_2. As shown in the main article text, reactions S1 and S2 are the rate-determining steps also of this second process, thus both PAA and H_2O_2 are degraded with similar kinetics (see Figure 1c in the main article). Here, we compare the kinetics of reactions S1 and S2 as a function of variable bromide and chloride concentrations.

Assume k' as the first-order rate constant of PAA degradation in reaction (S1), and k'' as the corresponding rate constant in reaction (S2). Then, assume $R = k'/k''$. Because $k' = k_1\ [Cl^-]$ and $k'' = k_2\ [Br^-]$, one gets the following trend for R vs. $[Cl^-]$ and $[Br^-]$:

$$R = \frac{k_1[Cl^-]}{k_2[Br^-]} \quad (S3)$$

Figure S3 reports the trend of R vs. $[Cl^-]$ and $[Br^-]$ according to Equation (S3).

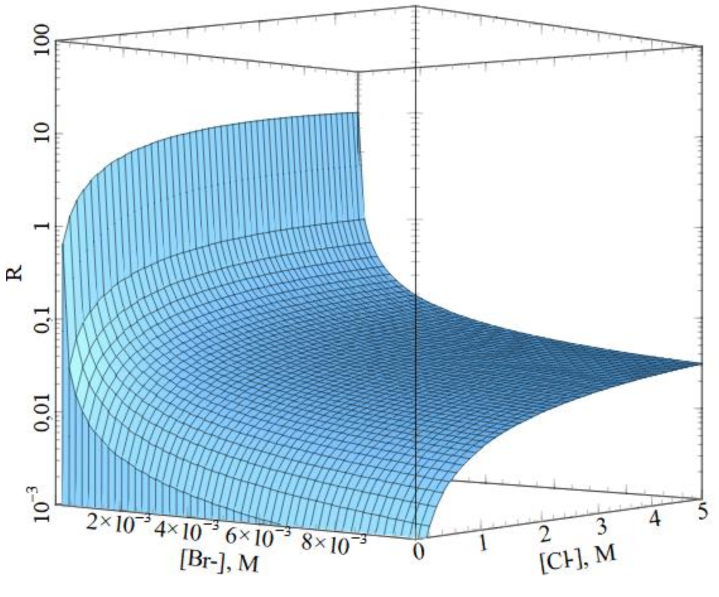

Figure S3. Trend of R vs. $[Cl^-]$ and $[Br^-]$ according to Equation (S3), in the bromide and chloride concentration ranges used as experimental conditions in the main article. Note the logarithmic scale in the vertical axis.

The trend shown in Figure S3 suggests the following:

(i) $R > 1$ (i.e., PAA is degraded by chloride faster than it is degraded by bromide) only when the bromide concentration is practically negligible;

(ii) If $[Br^-] \geq 1$ mM, it is not possible for chloride at the used concentrations (up to 5 M) to kinetically overcome bromide in the reaction with PAA.

These results suggest that the disappearance of PAA (and H_2O_2) observed experimentally in the presence of $[Cl^-] \geq 0.5$ M (see Figure 1b in the main article) was more likely due to small bromide impurities occurring in NaCl, rather than to chloride itself.

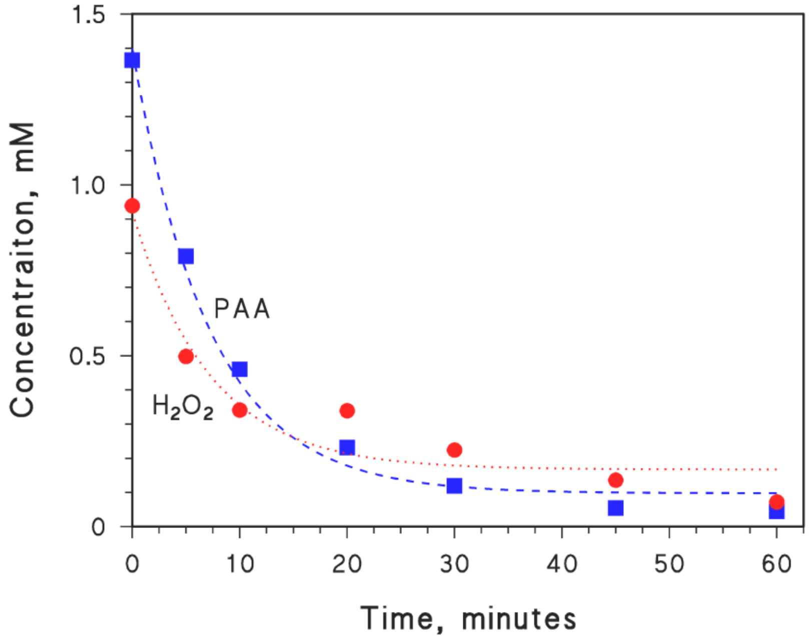

Figure S4. Fit of the experimental data of the time evolution of peracetic acid (PAA) and H_2O_2 (solid symbols: they are the same data as those shown in Figure 1c of the main article, in the presence of 10 mM KBr) with exponential functions having fixed a pseudo-first order degradation rate constant (k = 0.14 min^{-1}, derived from the kinetics of reaction S2). The residuals at high reaction times are probably due to the fact that the system becomes more complex as time passes, and can no longer be described by a simple pseudo first-order kinetic model.

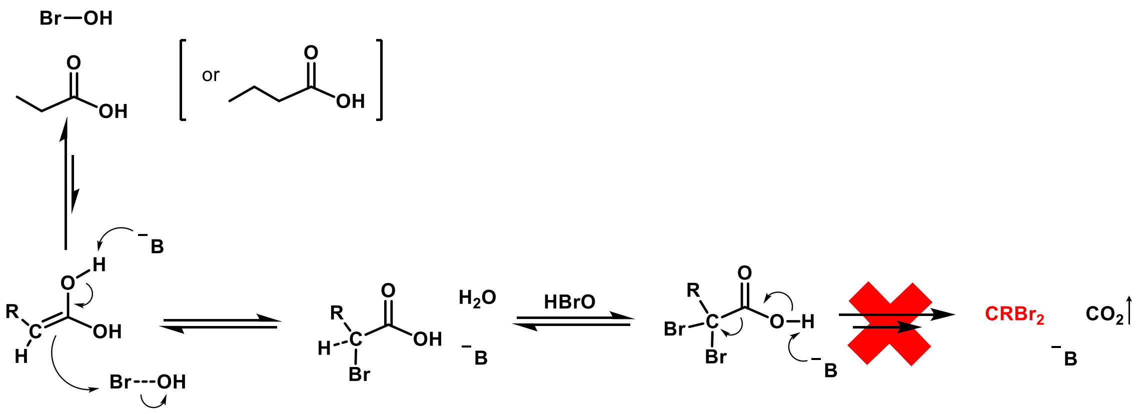

Scheme S1. According to a Hell-Vohlard-Zelinsky mechanism, the exit of the halogenated group is not promoted in the case of long chain carboxylic acids, since the groups are too nonpolar for an aqueous medium. Moreover, the formation of $CHBr_3$ is not allowed. B^- is intended as a generic base present in solution.

Table S1. Characteristics of the real contaminated groundwater.

Parameter	Units	Average Value or Range
Chloride	mg/L	2690
Bromide	mg/L	6.4
Fluoride	mg/L	220-320
Sulfate	mg/L	3130
Phosphate	mg/L	2400
Nitrate	mg/L	60
Bicarbonate	mg/L	10
N-NH_4^+	mg/L	480-590
TOC	mg^C/L	58 ± 12
Microalgae	cells/mL	$(1.76 \pm 0.6) \times 10^6$
pH		2.8

Table S2. List and quantification of halomethanes in the real contaminated groundwater before treatment.

	Parameter	U.M.	Value	LOQ
HACs	1,1,1-trichloroethane	µg/L	<LOQ	0.05
	1,1,2,2-tetrachloroethane	µg/L	<LOQ	0.05
	1,1,2-trichloroethane	µg/L	<LOQ	0.05
	1,1,1,2-tetrachloroethane	µg/L	<LOQ	0.05
	Trichloromethane	µg/L	<LOQ	0.05
	1,1-dichloroethane	µg/L	<LOQ	0.05
	1,1-dichloroetilene	µg/L	<LOQ	0.05
	1,1-dichloropropene	µg/L	<LOQ	0.05
	1,2,3-trichloropropane	µg/L	<LOQ	0.001
	1,2 -dibromoethane	µg/L	<LOQ	0.05
	1,2-dichloropropane	µg/L	<LOQ	0.05
	1,3-dichloropropane	µg/L	<LOQ	0.05
	Hexachlorobutadiene	µg/L	<LOQ	0.05
	Tetrachloroetilene	µg/L	<LOQ	0.05
	Trichloroetilene	µg/L	<LOQ	0.05
	Tetrachloromethane	µg/L	<LOQ	0.05
	Dibromomethane	µg/L	<LOQ	0.05
	Bromochloromethane	µg/L	<LOQ	0.05
	Dichloromethane	µg/L	<LOQ	0.05
	Bromomethane	µg/L	<LOQ	0.05
	Chloromethane	µg/L	<LOQ	0.05
	Dibromomethane	µg/L	<LOQ	0.05
	Bromomethane	µg/L	<LOQ	0.05
	Chloromethane	µg/L	<LOQ	0.05

**THMs**	Dibromochloromethane	µg/L	<LOQ	0.05
	Trichloromethane	µg/L	<LOQ	0.05
	Bromodichloromethane	µg/L	<LOQ	0.05
	Bromochloromethane	µg/L	<LOQ	0.05
	Tribromomethane	µg/L	<LOQ	0.05

Table S3. List and quantification of halomethanes in the real contaminated groundwater following treatment with PAA 1.5 mM.

	Parameter	U.M.	Value	LOQ
**HACs**	1,1,1-trichloroethane	µg/L	<LOQ	0.05
	1,1,2,2-tetrachloroethane	µg/L	<LOQ	0.05
	1,1,2-trichloroethane	µg/L	<LOQ	0.05
	1,1,1,2-tetrachloroethane	µg/L	<LOQ	0.05
	Trichloromethane	µg/L	<LOQ	0.05
	1,1-dichloroethane	µg/L	<LOQ	0.05
	1,1-dichloroetilene	µg/L	<LOQ	0.05
	1,1-dichloropropene	µg/L	<LOQ	0.05
	1,2,3-trichloropropane	µg/L	<LOQ	0.001
	1,2 -dibromoethane	µg/L	<LOQ	0.05
	1,2-dichloropropane	µg/L	<LOQ	0.05
	1,3-dichloropropane	µg/L	<LOQ	0.05
	Hexachlorobutadiene	µg/L	<LOQ	0.05
	Tetrachloroetilene	µg/L	<LOQ	0.05
	Trichloroetilene	µg/L	<LOQ	0.05
	Tetrachloromethane	µg/L	<LOQ	0.05
	Dibromomethane	µg/L	<LOQ	0.05
	Bromochloromethane	µg/L	<LOQ	0.05
	Dichloromethane	µg/L	<LOQ	0.05
	Bromomethane	µg/L	<LOQ	0.05
	Chloromethane	µg/L	**1.25**	0.05
	Dibromomethane	µg/L	<LOQ	0.05
	Bromomethane	µg/L	<LOQ	0.05
	Chloromethane	µg/L	<LOQ	0.05

**THMs**	Dibromochloromethane	µg/L	**0.28**	0.05
	Trichloromethane	µg/L	<LOQ	0.05
	Bromodichloromethane	µg/L	<LOQ	0.05
	Bromochloromethane	µg/L	<LOQ	0.05
	Tribromomethane	µg/L	**6.2**	0.05
